# Influence of Traditional Vanilla Curing on Its Physicochemical Properties and Aromatic Profile

**DOI:** 10.3390/foods14091652

**Published:** 2025-05-07

**Authors:** Isabel Janid Perez-Viveros, Sergio Erick García-Barrón, Blanca Elizabeth Hernández-Rodríguez, Ariadna Isabel Barrera-Rodríguez, Claudia Ariadna Acero-Ortega, Anastacio Espejel-García

**Affiliations:** 1 Posgrado en Ciencia y Tecnología Agroalimentaria, Departamento de Ingeniería Agroindustrial, Universidad Autónoma Chapingo, Km. 38.5, Carretera México-Texcoco, Texcoco 56230, Mexico; al24610075@chapingo.mx; 2 ESDAI, Universidad Panamericana, Augusto Rodin 498, Ciudad de México 03920, Mexico; cacero@up.edu.mx; 3 Departamento de Preparatoria Agrícola, Universidad Autónoma Chapingo, Km 38.5, Carretera México-Texcoco, Texcoco 56230, Mexico; bhernandezr@chapingo.mx (B.E.H.-R.); abarrerar@chapingo.mx (A.I.B.-R.)

**Keywords:** *Vanilla planifolia*, Rate-All-That-Apply (RATA), consumers, traditional food, vanilla processing

## Abstract

Vanilla is native to Mexico and has social-cultural and economic importance. It is sensory characteristics are developed during the curing process, which is associated with the region where it is carried out since the know-how of each locality is involved. In this sense, this work aimed to evaluate the influence of the curing process. Four different processes from four regions (SJA, SLP, CQ and EPM) were considered, taking into account two curing conditions. Additionally, sample control was considered. The moisture content, protein, ether extract, ash and pH were analyzed. The aromatic profile was evaluated by the RATA methodology and liking level. Except for ash content, the process influenced the other physicochemical parameters. The moisture contents of SLP and CQ samples from Period 1, as well as SLP samples from Period 2, comply with the current Mexican Standard. SJA vanilla was “slightly” accepted in both periods, surpassing the control sample. In contrast, the CQ sample was the least preferred. Thirty-five aromatic descriptors were generated. At the sensory level, a clear separation of vanillas was observed according to the type of curing. The attributes described included caramel, dry fruit, fruity, honey, maltol, rancid, sweet, tree bark, vanilla and ashes, which boosted the liking level. On the other hand, the descriptors chemical, moisture, dairy, spicy, wood and lipids had a negative effect, proving that these factors can alter the aromatic balance, giving an unpleasant smell and reducing preference. It was confirmed that the curing process influences physicochemical parameters, the aromatic profile and the liking level. However, it would be necessary to consider other variables.

## 1. Introduction

Vanilla (*Vanilla planifolia* Jacks Ex. Andrews), an orchid native to Mexico, is one of the most widely used flavors in the world [1,2]. This orchid is in demand by the food, pharmaceutical, perfumery and cosmetic industries [3,4]. It is considered the second most expensive spice in the world [5], due to its labor-intensive production and processing [6]. For its elaboration, it must undergo a curing process, which favors the formation of phenolic, aromatic, fatty acid and organic compounds that contribute to its aromatic profile, which may be associated with physicochemical, structural and morphological changes, as well as to the species used in the process, environmental factors and endophytic microorganisms, but mainly due to processing [3,6,7]. This process generally comprises four stages: blanching or slaughtering, fermentation (sweating), sun drying and conditioning [6,8,9,10]. However, it is important to note that this process may vary depending on the different techniques and conditions carried out in each region [3,10] resulting in a product with a characteristic physicochemical and aromatic profile.

In Mexico, the curing process is mostly carried out in a traditional manner, which has generated an important demand for Mexican vanilla, as its aromatic profile is more complete compared to other commercially available species [10]. In that sense, local knowledge has been key to the preservation of processing techniques and the quality of the final product. Integrating traditional knowledge with sensory science approaches [11], is fundamental, as its commercial value depends largely on its sensory attributes [12]. Therefore, it is important to understand how the curing process influences these variations. Although the literature provides valuable information on advances in understanding the effect of the curing process on phytochemical composition [13], the effect of different curing methods [5,14,15,16], as well as the biochemical and structural changes that vanilla undergoes during processing [17], and the effect on bacterial composition [9,18,19], it is evident that there are still areas of opportunity in scientific research. Particularly, there is little information on how the curing process influences the sensory profile, inasmuch as few studies have addressed the sensory characterization of vanilla.

For example, Franco et al. [20] conducted a sensory characterization of vanilla extracts from Brazilian regions. The impact of vanilla origin on the sensory characteristics of chocolate has also been evaluated [21]. Furthermore, the impact of the post-harvest period on the chemical and sensory properties of *Vanilla planifolia* and *Vanilla pompona* has been investigated [12]. Nowadays, sensory characterization can be carried out using methodologies that allow these characteristics to be evaluated efficiently, such as descriptive sensory analysis methods that simultaneously allow the acceptability of a product to be evaluated without the need for a trained panel [21,22]. An alternative is the RATA (Rate-All-That-Apply) methodology, which is a variant of CATA (Check-All-That-Apply) questions that has proven to be effective in differentiating products with similar sensory characteristics, but with subtle variations in the intensity of their attributes [23], as is the case with vanilla. In this method, consumers receive a predefined list of sensory attributes and must select those that they consider applicable to a specific product and evaluate their intensity [24]. Furthermore, it has been shown that when this methodology is applied in conjunction with a liking level test, it allows the identification of attributes that influence consumer preferences without bias and improves discrimination ability [25].

Hence, according to the literature review, there is little information about the sensory characteristics and physicochemical composition of vanilla. We believe that our study could make a great contribution, as the literature is limited in understanding the relationship between the curing process, the aromatic profile, the level of liking and the physicochemical composition of vanilla. Consequently, the purpose of this study was to characterize the aromatic profile of vanilla beans from four producing regions of Mexico traditionally processed according to the specific techniques of each area, considering two periods of sweat drying. In addition, the possible relationship between the liking level and its physicochemical composition was evaluated.

## 2. Materials and Methods

### 2.1. Vanilla Samples

**Figure 1:**
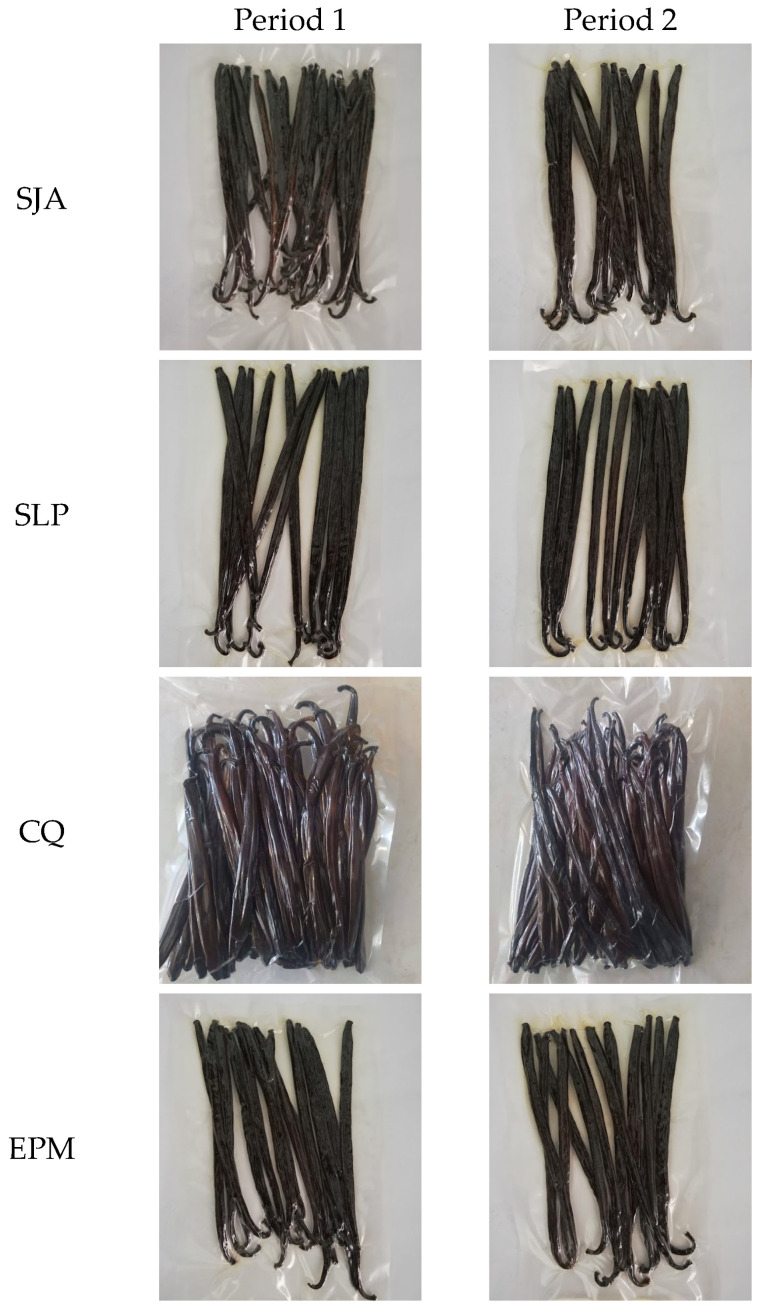
Vanilla samples with different sweating–drying periods used in the study.

Vanilla beans were collected from four different traditional processes: San José Acateno, Puebla (SJA); Jalpilla, San Luis Potosí (SLP); Cerro Quemado, Oaxaca (CQ); and Ejido Primero de Mayo (EPM), Veracruz, curing from 8 to 30 cycles, and the characteristics of the samples are summarized in Table 1. The samples were packed in a high vacuum (Figure 1) in a cool, dark place until analysis. It is important to note that a cycle corresponds to one day during which the pods are placed in the sun and then stored in sweatboxes [10]. Additionally, a commercial sample from the state of Puebla was used as a control for comparison; this sample was chosen because vanilla is traded in local markets and represents the standard conditions under which vanilla is traded. The curing process to which this sample was subjected was carried out in a traditional manner, including key stages such as manual harvesting, killing, drying and conditioning.

### 2.2. Physicochemical Analysis

Physicochemical determination was carried out using pods ground for 1 min in an electric mill model NB-201 201 (NUTRIBULLET^®^, Capital Brands, Los Angeles, CA, USA) with a power of 900 W. The analyses were conducted following current Mexican regulations, including moisture content [26], protein [27], ethereal extract by the Soxhlet method [28], ashes [29] and pH [30].

### 2.3. Ethical Aproval

Ethical approval for this study was obtained from the Agroindustrial Engineering Department, application 602.6/0541. The test protocol was reviewed and approved on 10 April 2024.

### 2.4. Sensory Evaluation Test

#### 2.4.1. Generation of Descriptors

To generate the descriptors, in a preliminary session, an open discussion was held with 10 of the total number of participants, who were experienced in sensory evaluation as mentioned by Franco et al. [20]. For this purpose, the samples were presented in pieces of approximately 0.05 g [12] in number 0 glasses covered with aluminum foil to avoid odor loss. The participants were instructed to smell each of the samples and to identify and write down on a sheet of paper those terms that allowed them to describe the vanilla beans. Subsequently, from the lists generated by the participants, the most frequently mentioned descriptors were chosen [31]. Furthermore, a literature review was made to look for some other descriptors that were not considered for inclusion and to conform to the final format that would be used for the RATA test.

#### 2.4.2. Participants

The study was conducted with 62 individuals aged between 18 and 66 years (68% women and 32% men). The selection criteria were to be over 18 years of age and to be available and interested in participating in the study. The tests were executed in the sensory analysis laboratory of the Center for Research and Assistance in Technology and Design of the State of Jalisco, A.C. (CIATEJ). Tests were carried out in a range of 22–25 °C.

#### 2.4.3. Liking Level Data and RATA Questions

The tests were carried out at two different sessions, one to evaluate the Period 1 pods and the other to evaluate the Period 2 pods on different days. During each session, participants evaluated four samples, plus a control sample. The pods were presented in pieces of approximately 1 cm, in number 0-type cups, coded with random three-digit numbers, and covered with aluminum foil to prevent odor loss. The order of sample presentation was randomized following a Latin square experimental design [32] to balance the order of presentation and minimize carryover effects. Each session was divided into two consecutive trials, with a 10 min rest period in between. Each consumer session began with a brief explanation of the test (5 min). First of all, participants were instructed to smell the samples and evaluate their liking level using a 9-point hedonic scale, ranging from “I dislike it very much” to “I like it very much” [20,33,34]. Subsequently, they were asked to answer RATA [20,24] questions based on the lexicon developed and described in Section 2.4.1. Participants were asked to smell and select the terms they considered appropriate to describe the object of study and to rate their intensity using a 9-point scale anchored between “low” (1) and “high” (9). This scale was designed to provide sufficient space for participants, allowing them to express differences between samples [35]. Seven different formats were developed for the participants, with the order of presentation of the descriptors also randomized. To minimize any carryover effect, they were asked to smell whole coffee beans between each sample [36].

### 2.5. Data Analysis

Analyses were performed within each period, during which vanilla samples were considered independent variables.

#### 2.5.1. Physicochemical Data

A one-way ANOVA was performed to evaluate significant differences in physicochemical variables between vanilla samples. The significance of differences between samples was determined using Tukey’s test (*p* < 0.05).

#### 2.5.2. Liking Level Data and RATA Questions

ANOVA was used to evaluate the presence of significant effects between samples on the mean global liking level. Subsequently, the significance of differences between samples was determined using Tukey’s test (*p* < 0.05).

The mean intensity was obtained for each attribute. RATA intensity scores were treated as continuous data [23,35]. A two-way ANOVA (with sample as a fixed factor and participant as a random factor) was conducted for all sensory descriptors within each period to analyze differences between pods in terms of descriptors intensity. Post hoc tests were performed with Tukey’s honest significant difference (HSD) test at a 95% confidence level in case of significant differences. A principal component analysis (PCA) was conducted using the mean intensity scores of descriptors, considering only the number of participants who decided that the attribute was applicable to describe the sample [24] and including supplementary quantitative variables such as liking level and instrumental data to examine existing relationships and increase the quality of the interpretation. All statistical analyses were performed in XLSTAT v. 2019 software.

## 3. Results

### 3.1. Physicochemical Analysi Results

Table 2 shows the results of the physicochemical composition expressed in percentages (dry weight).

Significant differences were observed in the parameters evaluated between vanilla samples in the two periods, confirming that the process carried out in each region influences physicochemical variables [10,17,37]. When pods were compared within Period 1, all variables showed significant differences (*p* < 0.05). In contrast, in Period 2, only the ash content showed no differences. In the case of moisture content, it was observed that the CQ and EPM samples in Period 1 had the same moisture content as the control sample. Whereas in Period 2, all samples were statistically different, with the control sample having the highest percentage. It is important to highlight that as the sweating–drying periods increased, the moisture level decreased, which is logical since one of the objectives during processing is to reduce the moisture content [8]. However, SJA vanilla had low moisture levels, which could indicate that the drying process was longer. In addition, it is important to mention that the SLP and CQ samples from Period 1 and the SLP sample from Period 2 comply with the Mexican standard [38]. This parameter is important because it affects aroma, flexibility and gloss. Moreover, it influences the preservation of the pod as a high percentage can lead to the development of microorganisms and have a negative effect [10,37,39]. On the contrary, if the humidity is low, it indicates that drying was prolonged, which caused the pod to lose flexibility and shine [8,40]. This can make the pod fragile and brittle, as was the case with the SJA sample from Period 2, which exhibited the lowest percentages, being below the optimal content [41]. Moisture variations in this study could be attributed to non-uniform drying, generated by differences in pod size, moisture content before processing, environmental conditions and relative humidity during sun drying, all of which can significantly impact the characteristics of the final product [8].

Regarding the content of ether extract in Period 1, clear differences are observed between the CQ and EPM samples while, in Period 2, the samples that differed were vanilla from SLP and SJA. However, the values found in this study are different from those reported by other authors [3,10]. These differences could be the result of a complex interaction between the physiological changes in the pods and the metabolic pathways that control the synthesis and degradation of macromolecules [42]. They could also be related to the origin of the vanilla [43] but are mainly due to the chemical changes that occur during the curing process, where certain enzymes that help in the breakdown of complex lipids are activated, releasing fatty acids such as oleic, palmitic and linoleic acid, which are precursors of compounds that contribute to the aroma of vanilla [44]. The results obtained coincide with those reported by Azeez [45] (pp. 287–311), who found that unprocessed vanilla contains 11% lipids. Therefore, it can be inferred that the lipid fraction increases after processing. As mentioned by Rao and Ravishankar [46], after processing, lipids tend to increase. This increase may be associated with the presence of oleoresins in the pod [42]. This confirms the importance of the curing process as a key factor affecting the composition of the ethereal fraction of vanilla. However, it should be noted that his behavior may also be due to other factors such as species and pod origin [37].

In the case of ash content, significant differences (*p* < 0.05) were observed among the samples in Period 1. On the contrary, no differences were observed in Period 2. It is relevant to note that, in both analyzed periods, the ash percentage ranged between 5 and 8%. According to Datta et al. [47] the ash content is proportional to the mineral composition of the fruit. The variations observed in this parameter could be due fruit genetic factors, growing conditions, agricultural practices, soil conditions and fertilization, as suggested by different studies [42,48,49]. Additionally, the drying method significantly influences fruit proximal composition except for ash content [48].

Regarding the protein content in Period 1, the SJA, CQ and EPM samples differed significantly from the control sample, showing lower protein content. Meanwhile, in Period 2, the control and SJA samples presented similar content. In contrast, the SLP, EPM and CQ samples had lower values. In that sense, Pérez-Silva et al. [42] and Peña-Barrientos et al. [50], mentioned that the protein content is between 4 and 5%. However, the SLP samples from Period 1, SJA from Period 2, and control in both periods presented higher percentages. The observed variations could be influenced by processing, since a series of enzymatic reactions are carried out during cellular decompartmentalization, specifically during the killing of the fruit, where protein denaturation takes place [42]. The protein fraction is crucial, as it plays an important role in biosynthetic and metabolic processes [17,51,52]. Many of these proteins act at the enzymatic level in the biosynthesis of aromatic compounds during the curing process, which is a type of fermentation that contributes significantly to the development of vanilla’s characteristic aroma [16,17,46,53].

In general, pH values ranged between 4.2 and 4.9, with significant differences (*p* < 0.05) between samples. This parameter is relevant, since various enzymatic reactions that influence aroma development and color occur during and after processing [16,54]. It plays an important role in vanilla metabolism; small decreases can affect the fruit’s sensory profile as well as create conditions for microbial growth, mainly of fungi [54]. The analysis results showed that the values were low, as it has been reported that a value below 5.5 is associated with low-quality vanilla [55]. This could be because β-glucosidase, the main enzyme responsible for the release of aromatic compounds, reaches its maximum activity at a pH of 6.5, but is unstable at a lower pH [54,56]. Probably, the low values observed in this study may have been influenced by the maturity stage of the pod [57] or microbial activity during processing [58]. To determine the relative composition of processed vanilla beans, it is necessary to take various factors into account. Although there is evidence that the practices carried out during processing influence the composition of beans, the influence of other factors such as the species and environmental factors to which the fruit is exposed during its growth, among others, should not be ruled out [3,46].

### 3.2. Sensory Evaluation Results

#### 3.2.1. Comparison of the Level of Liking Between Samples

The overall average scores of the liking level are shown in Table 3. According to Tukey’s test, no differences were observed between pods from different regions in the liking level. Except for the SJA sample (6.02), most of the samples were evaluated in the “neither like nor dislike” zone (5.24–5.52). In the case of Period 2, significant differences (*p* < 0.05) were observed between samples, indicating that there is an effect of the process carried out in the regions.

According to the results, it was observed that the higher the number of sweating–drying periods, the more participants were able to differentiate the samples according to their liking level. This shows that the process performed under different conditions has a significant impact on this measurement [33]. In addition, in both periods, the SJA sample was evaluated in the acceptance zone (between 6.0 and 6.5, “slightly”), indicating that the participants rated this vanilla sample positively, even better than the control sample, which obtained a rating of 5.2. This suggests that vanilla processed in this region could be valued even better than commercial vanilla. On the contrary, the CQ vanilla sample of Period 2 was the least liked, presenting lower values (4.3). Therefore, it would be convenient to analyze its processing to improve the level of consumer liking, even for samples that presented a neutral valoration.

#### 3.2.2. Rate-All-That-Apply (RATA) Results

According to the results, it was observed that the higher the number of sweating–drying periods, the more participants were able to differentiate the samples in line with their liking level. Table 4 and Table 5 summarize the results of the aromatic characterization of the vanilla beans analyzed using the RATA questions. A total of thirty-five terms were generated regardless of the process and number of periods of curing to which the pods were subjected, and the terms used were the same. These terms are present in Appendix A. Some of these descriptors are consistent with those identified in other studies [12,20,33,53]. Sensory characterization of vanilla beans has been reported by a panel of 50 evaluators who identified gourmand, vanilla, milky and spicy notes [12]. In another study, 64 consumers evaluated sweet, fruity, floral, spicy, fermented and sour notes [33]. In that sense, the vocabulary obtained shows the influence that the curing process has on the aromatic profile. This is especially relevant in the traditional process, where the pods are exposed to the sun, which considerably influences the generation of odors [12].

Significant differences (*p* < 0.05) were found in the intensity means for 20 of the 35 descriptors in Period 1, which showed the highest number of discriminating attributes. The differences in the intensity of more than half of the descriptors suggest that vanilla has a distinctive aromatic profile, thus contributing to a sensory identity of its own. For Period 2, significant differences were observed in 13 of the 35 descriptors between samples. This result could be because as the number of sweating–drying cycles increased, the descriptors became less discernible. One of the factors that could explain this behavior is that, during drying, volatile compounds decrease by approximately 30% due to evaporation from sun drying and/or water loss during pod transpiration [59]. This process could have influenced the fact that participants perceived fewer descriptors in Period 2. Importantly, participants did not perceive differences in the intensity of attributes such as sour, fruity, cooked, fresh, apple, spicy, pineapple and vanilla in both periods, suggesting that these descriptors could be considered characteristic of these pods, as they have also been reported in other studies [33,59,60].

The bitter attribute did not show variations in Period 1 but did in Period 2, although it was detected with low intensity. The caramel descriptor was more pronounced in EPM and CQ pods from P1, while in Period 2, it was only detected at a low intensity in the CQ pod. It is important to note that the chemical descriptor was perceived with high intensity in two of the samples, EPM and CQ, in both periods, especially in CQ in Period 2, which could have influenced the liking level, making this vanilla the least pleasant. The chocolate descriptor was identified with high intensity in two of the pods from Period 1, while in Period 2, although there were differences between samples, it was perceived as low. This note has been identified in vanilla from Puebla [60]. The dairy descriptor did not show differences in Period 1, but in Period 2, it was rated as intense in CQ vanilla. It is worth noting that the fermented note was rated with high intensity in CQ and SLP pods in both periods. According to Van Dyk et al. [33], the high scores of fermented odors could be due to the enzymes involved in the cellular respiration process not being inactivated during the fruit slaughter stage and producing compounds that contributed to this note. The honey note showed differences in Period 1; however, it was perceived as low intensity, while in Period 2, it was rated as low and at the same level among the vanillas. This note, common in *Vanilla planifolia*, is produced after processing ethyl 2-phenylacetate, which gives a honey-like note [21].

The maltol descriptor had the same intensity in the EPM, SAJ and control samples in Period 1, increasing in SAJ in Period 2. On the other hand, the moisture attribute was only perceived with high intensity in the SLP sample of Period 1. This descriptor could be associated with the moisture content of the samples [12]. However, in our case, in the vanilla that was identified with greater intensity (SLP of Period 1), its moisture content was not as high compared to other samples, where this descriptor was not very intense. The descriptor prune had low intensity in CQ of Period 1 and a high intensity in SJA and the control sample in Period 2. This descriptor has already been identified in cured vanilla [12]. Regarding the olive descriptor, it was perceived with high intensity in the CQ sample of Period 1, but to a lesser extent and without differences in Period 2. The raisin descriptor was detected with high intensity in Period 1, and no differences were observed between samples, while in Period 2, it was detected with high intensity except in the CQ sample.

The resin note in Period 1 was more intense in the EPM vanilla than in the CQ vanilla, while in Period 2, although there were differences, it was perceived with low intensity. On the other hand, it was found that the descriptor rum was perceived with high intensity in the control sample, sample EPM of Period 1, and all the pods of Period 2, except vanilla CQ, which would confirm the effect of the beneficiation process on the development of this note. The perception of the descriptor rum is likely related to how the process of beneficiation is carried out and that it becomes more intense throughout it [59]. The descriptor smoked showed low intensity in both periods, which is convenient since the significant presence of this note is an indicator of low quality [59].

Regarding the soil descriptor, it did not show differences in Period 1, but it did in Period 2, although at a low intensity. It is worth noting that the sweet note was identified with high intensity in most of the pods, except in SJA and CQ in Period 1 and Period 2, respectively. This could be associated with the maturity of the fruit, as more mature pods tend to exhibit a more intense sweet aroma [61], which is due in part to the higher concentrations of vanillin that accumulate [33]. Finally, the toasted attribute did not differ in intensity between samples from Period 1, but it did in Period 2, with the EPM sample being perceived with high intensity. It is important to highlight how the vanilla descriptor was detected with high intensity in most samples, with no difference between the two periods; this is appropriate since this note is characteristic of the processed pod [53,60]. The wood notes were differentiated in Period 1, being more intense in EPM and control, while in Period 2, CQ showed greater intensity. The intensity of this note could be related to low-level liking of the sample, as mentioned by Brunschwig et al. [62], who indicated that if the wood notes are very pronounced, it could be considered a low-quality vanilla. Another important finding to mention is how in the CQ vanilla, the notes of olive, caramel and sweetness were perceived as intense in Period 1, and as the sweating–drying period increased, their intensity decreased. In contrast, the lactic, wood and chemical notes increased in intensity with processing time. This shows that, despite the vanilla beans being processed in the same way, the processing time affected the intensity with which the descriptors were perceived.

**Figure 2:**
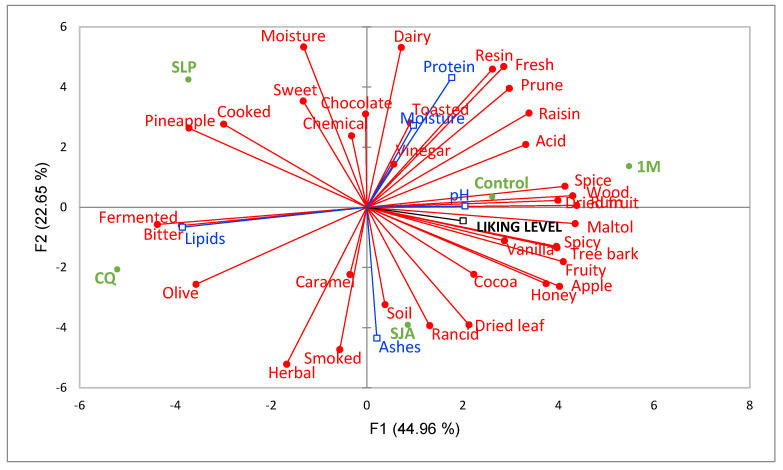
PCA based on the mean intensity scores of the RATA test aromatic descriptors used to describe the vanilla samples and supplementary variables: liking level and physicochemical parameters. (Colour green: samples; red: descriptors and blue: physicochemical parameters).

Finally, PCA was carried out to illustrate the relationships between the physicochemical variables and intensity means of descriptors. In the case of the mean score for the liking level, they were considered as supplementary variables. Figure 2, corresponding to Period 1, shows that with two dimensions, 67.6% of the variation in the data was explained. PC1 explained most of the variance (44.9%) and the second component (PC2) explained 22.6%. A clear separation of the pods according to the transformation process was observed. The sample positively correlated with PC1 was EPM, characterized by the highest number of descriptors (acid, apple, dried fruit, fruity, honey, maltol, prune, raisin, rum spice, spicy, tree bark and wood). According to Brunschwig et al. [62], the rum note is related to the process as it becomes more intense during processing. The CQ sample correlated negatively with PC1 and showed an association with the notes of bitter, cooked, fermented, olive and pineapple and physicochemical variable lipids. On the other hand, vanilla SLP correlated positively with PC2, characterized by the descriptors dairy, fresh, humidity and resin and with the physicochemical variable protein.

**Figure 3:**
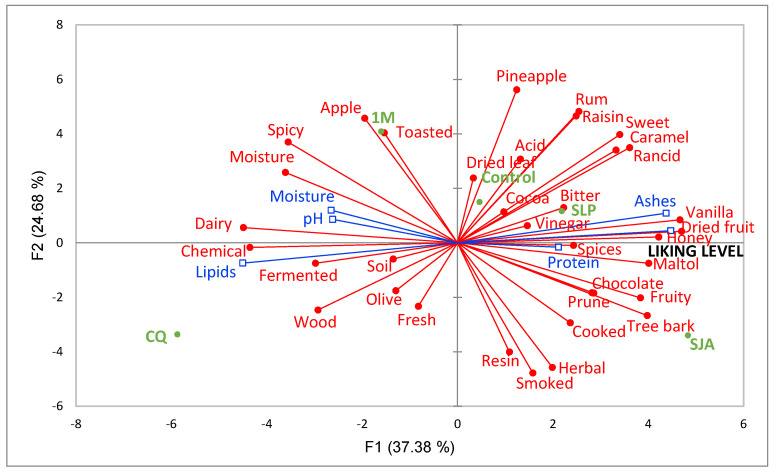
PCA based on the mean intensity scores of the RATA test aromatic descriptors used to describe the vanilla samples and supplementary variables: liking level and physicochemical parameters. (Colour green: samples; red: descriptors and blue: physicochemical parameters).

Concerning Period 2, Figure 3 shows that two dimensions explain 62% of the total variance. PC1 explained most of the variance (37.38%) and the second component (PC2) explained 24.68%. A clear separation of the pods according to the transformation process was observed.

The sample positively correlated with PC1 was SJA, showing a positive association with the descriptors caramel, fruity, honey, maltol, nuts, rancid, sweet, tree bark and vanilla and with the liking level, thus revealing that these descriptors were the ones that drove the liking level for this vanilla. This also agrees with Franco et al. [20], who suggested that sweet and vanilla notes are the desired characteristics that drive the liking level, while Takahashi et al. [63] found that nutty and sweet notes are positive factors for aroma. In contrast, the CQ sample correlated negatively with PC1 and showed a relationship with the chemical, humidity, dairy, spicy and woody notes and the lipid variable. Perhaps the relationship between lipids and lactic notes is due to the presence of aliphatic acids, since in a study by Pérez-Silva et al. [64], the presence of compounds such as valeric acid generated cheesy aromatic notes.

In addition, it was found that this sample was positioned opposite the liking level, which indicates that the presence of these factors can alter the aromatic balance, giving it an unpleasant odor that reduces the preference of participants. The descriptors identified for this period agree with those reported in previous works, where it has been mentioned that attributes perceived in pods include descriptors such as floral, sweet, spicy, tobacco-like, vanilla and woody [12]. This was also demonstrated in the work of Franco et al. [20], who identified vanilla and sweet notes in *V. planifolia*, the dominant species in Mexico.

On the other hand, PC2 showed a positive association with EPM vanilla, which was characterized by the descriptors apple, pineapple, raisin, rum and toasted, but was negatively correlated with the descriptors herbal, resin and smoky. Although vanilla processing is complex and involves many more variables [33], with RATA methodology, it was possible to discriminate between vanilla beans. The descriptors analyzed in this study were perceived differently in both periods of sweating–drying, and we attribute this to the production process influencing the aromatic profile while also affecting the liking level and physicochemical composition. However, according to the literature, many other factors can influence vanilla composition, such as harvest period, growing conditions, region and species [12,61]. Sensory characterization of traditional products such as vanilla can benefit production areas by helping us better understand the effect that processing has on the chemical and sensory properties of the pod, which have an effect on the economic development of communities, as well as help maintain forest areas [20] and conserve the traditional knowledge of Indigenous communities.

## 4. Conclusions

This study characterized the aromatic profile of traditionally processed vanilla using RATA sensory tests and physicochemical analysis. To our knowledge, this is the first work that sensorially and physically evaluated traditionally cured vanilla in two processing times, related physicochemical variables with the level of liking and compared this with commercial vanilla. The curing process significantly influenced physicochemical properties. The CQ samples in Period 1 and the SLP vanilla in both periods complied with the Mexican standard specification.

It was observed that the SJA samples had a higher liking, even surpassing the control sample, which suggests that vanilla from this region can compete in the market and reveals the importance of the curing process in the consumer’s perception.

Through the RATA test, olfactory descriptors were generated that varied in intensity and were useful to discriminate pods with similar sensory characteristics. In the PCA, samples were separated according to the transformation process, with pleasant descriptors such as caramel, nuts, fruity, honey, maltol, rancid, sweet, tree bark and vanilla standing out. The CQ vanilla had lower acceptance due to undesirable descriptors such as humidity, dairy, wood, spices and chemical and was associated with variable lipids, which shows that certain factors can deteriorate the perception of the aromatic profile, reducing consumer preference.

The differences in the variables evaluated are mainly due to the production process in each region, although other factors may also play a role. This work expands the literature on vanilla by investigating its sensory profile, physicochemical composition and level of liking, contributing to economic development and knowledge conservation in Indigenous communities. For future research, the use of gas chromatography is recommended to further investigate the role of volatile compounds in the sensory profile of vanilla.

## Figures and Tables

**Table 1:** Characteristics of the vanilla samples used in this study.

Samples	Variety	Area of Production	Date Harvested	Periods of Curing
SJA	*Vanilla planifolia*	San José Acateno, Puebla	2020	8 and 30 cycles
SLP	*Vanilla planifolia*	Cerro Quemado, Oaxaca	2020	8 and 30 cycles
CQ	*Vanilla planifolia*	Jalpilla, San Luis Potosí	2020	8 and 30 cycles
EPM	*Vanilla planifolia*	Primero de Mayo, Veracruz	2020	8 and 30 cycles
Control	*Vanilla planifolia*	Puebla	2020	N.A. *

* N.A.: Not Applicable.

**Table 2:** Physicochemical composition (g/100 g) of vanilla with different sweating–drying periods.

Sample	Period	Moisture	Ether Extract	Ashes	Protein	pH
Control	Period 1	41.02 ± 0.40 ^a^	12.98 ± 1.22 ^ab^	7.43 ± 0.15 ^a^	7.90 ± 0.82 ^a^	4.69 ± 0.01 ^c^
SLP		31.44 ± 0.70 ^c^	13.13 ± 0.61 ^ab^	5.45 ± 0.20 ^c^	6.78 ± 0.67 ^ab^	4.74 ± 0.02 ^b^
SJA		21.06 ± 0.96 ^c^	11.63 ± 0.79 ^bc^	6.99 ± 0.79 ^ab^	4.73 ± 0.34 ^cd^	4.93 ± 0.01 ^a^
CQ		36.61 ± 0.99 ^ab^	15.00 ± 0.86 ^a^	6.66 ± 0.91 ^abc^	4.47 ± 0.43 ^d^	4.31 ± 0.01 ^d^
EPM		39.04 ± 4.17 ^a^	10.04 ± 0.84 ^c^	5.55 ± 0.20 ^bc^	6.21 ± 0.32 ^bc^	4.66 ± 0.01 ^c^
Control	Period 2	41.02 ± 0.40 ^a^	12.98 ± 1.22 ^ab^	7.43 ± 0.15	7.90 ± 0.82 ^a^	4.69 ± 0.01 ^b^
SLP		31.02 ± 2.14 ^b^	16.56 ± 1.36 ^a^	6.75 ± 0.40	4.57 ± 1.06 ^b^	4.78 ± 0.02 ^b^
SJA		13.43 ± 0.23 ^e^	10.15 ± 0.79 ^b^	7.70 ± 0.67	6.10 ± 0.48 ^ab^	4.61 ± 0.03 ^c^
CQ		16.85 ± 0.62 ^d^	12.61 ± 0.06 ^ab^	7.30 ± 0.87	5.10 ± 0.90 ^b^	4.26 ± 0.02 ^d^
EPM		24.10 ± 0.21 ^c^	12.74 ± 2.61 ^ab^	7.34 ± 0.67	4.34 ± 1.06 ^b^	4.94 ± 0.04 ^a^

Samples: San José Acateno, Puebla (SJA); Jalpilla, San Luis Potosí (SLP); Cerro Quemado, Oaxaca (CQ); Ejido Primero de Mayo (EPM); Veracruz and commercial sample (control). Period: P1—8 and P2—30 sweating–drying periods. Each sweating–drying period corresponds to a drying phase in the sun and sweating that is carried out alternately and equivalent to one day. Data are represented as mean ± SD (*n* = 3). Different letters in each column for period indicate statistical differences (*p* < 0.05) according to Tukey’s test.

**Table 3:** Mean scores of the liking level of traditionally cured vanilla beans with different sweating–drying periods (*n* = 62).

Samples	Period 1	Period 2
SJA	6.02 ± 2.02	6.39 ± 1.45 ^a^
EPM	5.52 ± 2.01	5.52 ± 1.88 ^ab^
CQ	5.37 ± 2.06	4.34 ± 2.27 ^c^
SLP	5.24 ± 2.18	5.61 ± 1.91 ^ab^
Control	5.27 ± 2.05	5.27 ± 2.05 ^bc^

Data are represented as the average of ± SD. Means values with different letters within the same column do significantly differ (*p* ≤ 0.05) according to Tukey’s test.

**Table 4:** Means score of the Rate-All-That-Apply (RATA) test descriptors (Period 1).

Descriptor	EPM	SJA	CQ	SLP	Control	*p*-Value
Acid	5.46 ± 2.46	3.88 ± 3.00	4.00 ± 1.26	4.17 ± 2.93	5.86 ± 1.57	NS
Apple	4.60 ± 2.51	4.17 ± 2.22	2.33 ± 0.51	1.60 ± 0.54	3.71 ± 2.21	NS
Bitter	3.10 ± 1.10	3.91 ± 2.16	4.91 ± 2.73	4.50 ± 2.81	3.27 ± 1.95	NS
Caramel	6.59 ± 1.29 ^ab^	5.24 ± 1.99 ^bc^	7.47 ± 0.99 ^a^	4.69 ± 2.44 ^c^	5.47 ± 1.26 ^bc^	0.0001
Chemical	6.50 ± 1.51	3.75 ± 2.76	6.22 ± 2.27	5.29 ± 2.84	4.55 ± 3.47	NS
Chocolate	5.94 ± 1.18 ^a^	4.32 ± 2.26 ^b^	3.46 ± 1.26 ^bc^	6.23 ± 1.09 ^a^	1.86 ± 0.77 ^c^	0.0001
Cocoa	4.29 ± 1.89 ^a^	4.26 ± 1.85 ^a^	2.53 ± 1.24 ^b^	2.44 ± 0.81 ^b^	1.92 ± 0.66 ^b^	0.0001
Cooked	2.40 ± 2.07	1.80 ± 0.83	3.60 ± 2.41	3.33 ± 1.52	3.00 ± 2.00	NS
Dairy	4.00 ± 2.58	2.88 ± 1.55	3.00 ± 1.67	4.00 ± 1.41	3.00 ± 1.30	NS
Dried fruit	6.22 ± 1.30 ^a^	4.29 ± 2.39 ^bc^	3.00 ± 1.47 ^c^	3.00 ± 1.09 ^c^	5.33 ± 1.04 ^ab^	0.0001
Dried leaf	2.67 ± 1.07 ^ab^	3.20 ± 1.93 ^ab^	2.73 ± 1.84 ^ab^	1.94 ± 0.92 ^b^	3.63 ± 1.89 ^a^	0.043
Fermented	2.38 ± 1.02 ^c^	4.75 ± 2.43 ^b^	7.86 ± 1.09 ^a^	6.33 ± 1.23 ^ab^	6.05 ± 1.71 ^b^	0.0001
Fresh	6.00 ± 2.55	3.80 ± 2.30	2.60 ± 1.51	5.5 ± 3.42	5.38 ± 1.68	NS
Fruity	4.53 ± 2.47	3.89 ± 2.22	3.10 ± 1.59	2.71 ± 1.11	3.71 ± 1.89	NS
Herbal	2.30 ± 1.16	3.54 ± 2.02	3.33 ± 1.23	2.62 ± 1.04	3.10 ± 1.91	NS
Honey	5.63 ± 0.91 ^a^	4.48 ± 2.22 ^a^	3.25 ± 1.48 ^ab^	1.90 ± 0.87 ^b^	3.86 ± 2.59 ^ab^	0.002
Maltol	6.14 ± 1.67 ^a^	4.73 ± 2.15 ^ab^	3.17 ± 1.16 ^ab^	3.20 ± 1.13 ^b^	4.73 ± 2.15 ^ab^	0.019
Moisture	4.17 ± 2.32 ^b^	2.25 ± 1.48 ^b^	2.93 ± 1.71 ^b^	6.78 ± 1.20 ^a^	2.20 ± 0.78 ^b^	0.0001
Pineapple	2.14 ± 0.90	2.14 ± 1.06	4.00 ± 3.24	4.17 ± 2.40	3.22 ± 1.56	NS
Prune	6.88 ± 1.40 ^a^	5.19 ± 2.20 ^b^	2.64 ± 1.49 ^c^	6.60 ± 1.29 ^ab^	6.61 ± 1.33 ^a^	0.0001
Olive	2.88 ± 1.35 ^bc^	4.67 ± 2.34 ^b^	7.46 ± 1.36 ^a^	4.27 ± 2.90 ^bc^	1.86 ± 1.06 ^c^	0.0001
Raisin	6.53 ± 1.12	5.76 ± 2.30	5.27 ± 2.42	6.10 ± 1.19	6.83 ± 1.75	NS
Rancid	5.11 ± 2.61 ^a^	4.14 ± 2.11 ^ab^	5.09 ± 2.70 ^a^	1.78 ± 1.09 ^b^	3.40 ± 1.45 ^ab^	0.005
Resin	6.00 ± 1.26 ^a^	3.60 ± 1.43 ^ab^	2.75 ± 0.96 ^b^	5.13 ± 2.74 ^a^	4.15 ± 1.99 ^ab^	0.006
Rum	6.11 ± 1.26 ^a^	3.86 ± 2.10 ^b^	3.33 ± 1.72 ^b^	2.86 ± 1.06 ^b^	6.14 ± 1.29 ^a^	0.0001
Smoked	2.90 ± 0.99 ^ab^	3.88 ± 1.93 ^a^	3.30 ± 1.41 ^ab^	2.33 ± 1.23 ^b^	1.89 ± 0.78 ^b^	0.009
Soil	2.11 ± 1.26	3.46 ± 2.84	1.92 ± 1.24	2.29 ± 1.26	1.92 ± 0.76	NS
Spices	6.21 ± 1.57 ^a^	3.23 ± 2.18 ^b^	2.60 ± 1.35 ^b^	2.36 ± 1.15 ^b^	5.27 ± 1.61 ^a^	0.0001
Spicy	3.71 ± 2.36	3.43 ± 2.07	2.86 ± 1.21	2.67 ± 1.93	4.00 ± 2.65	NS
Sweet	6.77 ± 1.19 ^a^	4.89 ± 2.11 ^b^	7.20 ± 1.15 ^a^	6.77 ± 1.19 ^a^	6.43 ± 1.53 ^b^	0.0001
Toasted	2.75 ± 1.06	3.42 ± 1.92	2.94 ± 1.51	4.05 ± 2.32	4.00 ± 2.20	NS
Tree bark	4.68 ± 1.93	4.54 ± 2.04 ^ab^	2.88 ± 1.02 ^c^	2.92 ± 1.16 ^bc^	5.33 ± 1.07 ^a^	0.0001
Vanilla	6.36 ± 2.28	6.20 ± 2.33	5.47 ± 2.63	5.63 ± 2.24	5.46 ± 2.22	NS
Vinegar	7.00 ± 1.11 ^a^	4.44 ± 2.65 ^ab^	4.57 ± 3.18 ^ab^	5.13 ± 2.90 ^ab^	1.89 ± 0.78 ^b^	0.002
Wood	6.75 ± 1.48 ^a^	3.97 ± 2.17 ^b^	2.67 ± 1.35 ^c^	2.63 ± 1.14 ^bc^	5.83 ± 1.55 ^a^	0.0001

Data are represented as the average of ± SD. Means values with different letters within the same row do significantly differ (*p* ≤ 0.05) according to Tukey’s test. NS: not significant.

**Table 5:** Means score of the Rate-All-That-Apply (RATA) test descriptors (Period 2).

Descriptor	EPM	SJA	CQ	SLP	Control	*p*-Value
Acid	4.70 ± 1.49	4.25 ± 0.50	3.30 ± 1.82	3.71 ± 2.62	5.86 ± 1.57	NS
Apple	4.00 ± 2.45	2.50 ± 2.34	3.00 ± 1.41	2.75 ± 2.22	3.71 ± 2.21	NS
Bitter	3.18 ± 1.77 ^ab^	3.40 ± 2.51 ^ab^	2.90 ± 1.66 ^b^	5.38 ± 1.30 ^a^	3.27 ± 1.95 ^ab^	0.056
Caramel	6.50 ± 1.09 ^a^	6.10 ± 1.29 ^a^	2.79 ± 1.42 ^b^	6.00 ± 1.41 ^a^	5.47 ± 1.26 ^a^	0.0001
Chemical	7.00 ± 1.41 ^ab^	4.14 ± 2.97 ^b^	7.93 ± 0.82 ^a^	3.90 ± 2.80 ^b^	4.55 ± 3.47 ^b^	0.0001
Chocolate	2.56 ± 1.01 ^ab^	3.97 ± 2.44 ^a^	2.64 ± 1.50 ^ab^	4.24 ± 2.16 ^a^	1.86 ± 0.77 ^b^	0.002
Cocoa	4.13 ± 1.64 ^a^	3.62 ± 2.30 ^ab^	2.80 ± 1.31 ^ab^	3.58 ± 2.57 ^ab^	1.91 ± 0.66 ^b^	0.044
Cooked	2.67 ± 1.52	3.14 ± 1.54	3.00 ± 1.63	3.29 ± 2.49	3.00 ± 2.00	NS
Dairy	4.75 ± 2.87 ^ab^	1.80 ± 0.83 ^b^	6.00 ± 2.94 ^a^	3.75 ± 0.95 ^ab^	3.00 ± 1.30 ^ab^	0.026
Dried fruit	4.44 ± 2.31 ^ab^	6.08 ± 1.49 ^a^	3.25 ± 1.48 ^b^	5.55 ± 1.21 ^a^	5.33 ± 1.04 ^ab^	0.003
Dried leaf	3.00 ± 1.85 ^b^	2.00 ± 0.66 ^b^	3.00 ± 1.37 ^b^	5.82 ± 1.07 ^a^	3.63 ± 1.89 ^b^	0.0001
Fermented	3.00 ± 1.82 ^c^	2.00 ± 0.86 ^c^	7.62 ± 1.19 ^a^	6.08 ± 1.56 ^ab^	6.05 ± 1.71 ^b^	0.0001
Fresh	3.00 ± 1.78	4.00 ± 2.44	4.75 ± 2.87	3.83 ± 2.25	5.38 ± 1.68	NS
Fruity	3.90 ± 2.80	5.82 ± 1.16	3.20 ± 2.04	3.82 ± 2.35	3.71 ± 1.89	NS
Herbal	2.17 ± 1.03 ^b^	4.00 ± 2.09 ^a^	3.00 ± 1.61 ^ab^	2.38 ± 0.51 ^ab^	3.10 ± 1.91 ^ab^	0.048
Honey	4.21 ± 2.52	5.24 ± 1.34	2.80 ± 1.47	3.95 ± 2.24	3.86 ± 2.59	NS
Maltol	4.80 ± 1.75 ^ab^	7.00 ± 2.00 ^a^	2.85 ± 1.34 ^b^	4.31 ± 1.93 ^b^	4.09 ± 2.38 ^b^	0.001
Moisture	2.21 ± 1.62	1.67 ± 0.65	2.14 ± 1.21	1.75 ± 0.70	2.20 ± 0.78	NS
Pineapple	4.13 ± 3.09	2.25 ± 0.95	1.50 ± 0.70	3.40 ± 2.61	3.22 ± 1.56	NS
Prune	2.00 ± 0.93 ^b^	6.73 ± 1.28 ^a^	2.4 ± 1.35 ^b^	2.40 ± 1.35 ^b^	6.61 ± 1.33 ^a^	0.0001
Olive	3.90 ± 2.07	3.75 ± 1.90	3.67 ± 1.65	2.00 ± 0.89	1.86 ± 1.06	0.044
Raisin	6.88 ± 1.45 ^a^	5.53 ± 1.66 ^a^	3.23 ± 1.64 ^b^	5.81 ± 1.51 ^a^	6.83 ± 1.75 ^a^	0.0001
Rancid	3.64 ± 2.33	3.50 ± 2.07	2.80 ± 1.31	4.00 ± 2.13	3.40 ± 1.45	NS
Resin	1.82 ± 0.87 ^b^	4.50 ± 2.55 ^a^	3.50 ± 1.22 ^ab^	2.00 ± 0.92 ^b^	4.15 ± 1.99 ^a^	0.001
Rum	6.00 ± 1.09 ^a^	4.75 ± 0.70 ^a^	2.93 ± 1.22 ^b^	5.80 ± 1.61 ^a^	6.14 ± 1.29 ^a^	0.0001
Smoked	2.14 ± 1.34 ^ab^	3.88 ± 2.36 ^a^	2.85 ± 1.46 ^ab^	2.22 ± 0.66 a^b^	1.89 ± 0.78 ^b^	0.023
Soil	2.36 ± 1.36 ^ab^	2.09 ± 0.94 ^ab^	3.22 ± 1.35 ^ab^	3.73 ± 2.5 ^a^	1.92 ± 0.76 ^b^	0.015
Spices	2.56 ± 1.20 ^b^	3.95 ± 2.24 ^ab^	2.81 ± 1.51 b	3.65 ± 2.56 ^ab^	5.27 ± 1.61 ^a^	0.007
Spicy	4.50 ± 2.44	1.00 ± 0	3.80 ± 1.64	2.80 ± 1.78	4.00 ± 2.65	NS
Sweet	6.80 ± 1.32 ^a^	6.19 ± 1.44 ^a^	3.23 ± 1.34 ^b^	6.32 ± 1.64 ^a^	6.43 ± 1.53 ^a^	0.0001
Toasted	6.10 ± 1.28 ^a^	2.64 ± 0.50 ^bc^	3.00 ± 1.73 ^bc^	2.08 ± 0.76 ^c^	4.00 ± 2.20 ^b^	0.0001
Tree bark	2.78 ± 1.06 ^c^	6.93 ± 1.28 ^a^	3.65 ± 1.16 ^c^	5.62 ± 1.32 ^b^	5.33 ± 1.07 ^b^	0.0001
Vanilla	5.35 ± 2.39	5.88 ± 2.28	4.70 ± 2.55	5.55 ± 2.21	5.46 ± 2.22	N.S.
Vinegar	2.75 ± 1.48	2.75 ± 0.95	2.50 ± 1.58	4.14 ± 2.97	1.89 ± 0.78	N.S.
Wood	6.77 ± 1.30 ^ab^	6.58 ± 1.50 ^abc^	7.33 ± 0.90 ^a^	5.41 ± 1.59 ^c^	5.83 1.55 ^bc^	0.001

Data are represented as the average of ± SD. Means values with different letters within the same row do significantly differ (*p* ≤ 0.05) according to Tukey’s test. NS: not significant.

## Data Availability

The original contributions presented in this study are included in the article. Further inquiries can be directed to the corresponding author.

## Appendix A

**Table A1:** Aromatic lexicon development for participants (*n* = 62).

	Aromatic Descriptor	Frequency (Total)
1	Wood	515
2	Raisins	384
3	Rum	239
4	Prune	429
5	Apple	99
6	Toasted	306
7	Vinegar	159
8	Dairy	85
9	Herbal	241
10	Rancid	166
11	Bitter	197
12	Sweet	556
13	Spicy	369
14	Vanilla	747
15	Chemical	177
16	Smoked	254
17	Fresh	134
18	Honey	303
19	Cocoa	287
20	Chocolate	363
21	Moisture	243
22	Dried Fruit	324
23	Fermented	318
24	Pineapple	96
25	Soil	243
26	Olive	168
27	Tree Bark	365
28	Acid	140
29	Dried Leaf	325
30	Maltol	192
31	Spicy	115
32	Resin	210
33	Fruity	252
34	Cooked	87
35	Caramel	421
